# The relationship between equol production status and normal tension glaucoma

**DOI:** 10.1007/s10792-024-03225-3

**Published:** 2024-06-27

**Authors:** Noriko Himori, Keiko Uchida, Takahiro Ninomiya, Masashi Nagai, Kota Sato, Satoru Tsuda, Kazuko Omodaka, Toru Nakazawa

**Affiliations:** 1 Department of Ophthalmology, Tohoku University Graduate School of Medicine, 1-1 Seiryo-cho Aoba-ku, Sendai, Miyagi 980-8574 Japan; 2 Department of Aging Vision Healthcare, Tohoku University Graduate School of Biomedical Engineering, Tohoku University, Sendai, Japan; 3 Healthcare Systems Co., Ltd, Nagaya, Japan; 4 Department of Ophthalmic Imaging and Information Analytics, Tohoku University Graduate School of Medicine, Sendai, Japan; 5 Department of Retinal Disease Control, Tohoku University Graduate School of Medicine, Sendai, Japan; 6 Department of Advanced Ophthalmic Medicine, Tohoku University Graduate School of Medicine, Sendai, Japan

**Keywords:** Glaucoma, Equol, Female hormone, Lifestyle habits, Soy isoflavone

## Abstract

**Purpose:**

Equol is metabolized by intestinal bacteria from soy isoflavones and is chemically similar to estrogen. Dietary habits, such as consumption of soy products, influence equol production. A relationship between glaucoma and estrogen has been identified; here, we investigated the relationship between equol production status and glaucoma in Japan.

**Methods:**

We recruited 68 normal-tension glaucoma (NTG) patients (male to female ratio 26:42, average age 63.0 ± 7.6 years) and 31 controls (male to female ratio 13:18, average age 66.0 ± 6.3 years) from our hospital. All women included were postmenopausal. Urinary equol concentration was quantified with the ELISA method. MD was calculated based on the Humphrey visual field. The association between MD and equol was analyzed with Spearman’s rank correlation coefficient. The Mann–Whitney U test was used to compare the equol-producing (> 1 μM) and non-producing (< 1 μM) subjects. We also investigated the association between equol and glaucoma with a logistic regression analysis.

**Results:**

There was a significant association between equol and MD (r = 0.36, *P* < 0.01) in the NTG patients. Glaucoma, represented by MD, was significantly milder in the equol-producing subjects than the non-equol producing subjects (*P* = 0.03). A multivariate analysis revealed the independent contributions of equol, cpRNFLT, and IOP to MD (*P* = 0.03, *P* = 0.04, and *P* < 0.01, respectively).

**Conclusion:**

Our results suggest that equol, acting through estrogen receptor-mediated neuroprotective effects, might be involved in suppressing the progression of NTG. This result also adds to evidence that glaucoma may be influenced by lifestyle.

## Introduction

Equol is a highly active soy isoflavone. Soy isoflavones include 3 compounds: daidzein, genistein, and glycitein. Equol is generated from daidzein in soybeans by equol-generating enterobacteria, but not every person produces equol in this way. It has been reported that there are more equol producers in Japan than in Western countries because of dietary habits [1, 2]. It is also known that Japanese equol producers are decreasing in number due to Westernization of the diet in younger generations and a decrease in the amount and frequency of soy food intake and the dietary fiber intake necessary for the maintenance of an equol-producing intestinal environment.

It has been reported that equol activates nuclear-factor erythroid 2–related factor 2 (Nrf2) [3], that it has anti-inflammatory effects [4], and that it inhibits collagen degradation [5]. These effects may be due to equol having a structure similar to that of estrogen. There are three kinds of estrogen receptors: α, β, and G protein-coupled. Equol is known to have a high affinity with estrogen receptor β. Equol has an estrogen-like effect that is 1/1000th to 1/100th as strong as that of estrogen. Epidemiological studies have studied the association between health outcomes and the intake of soy foods. Reported benefits of being an equol producer include a lowered risk of lifestyle-related disease [6, 7] and cancer [8, 9]. Thus, it would be useful to determine whether individuals are equol producers or not.

Glaucoma is the second most common cause of blindness in the world [10]. Some research has revealed associations between glaucoma and female hormones. The Rotterdam Study showed that women had a 2.6-fold increased risk of glaucoma when they underwent menopause before they were 45 years old [11]. Furthermore, adequate estrogen replacement therapy reduces the risk of glaucoma in women [12]. Finally, estrogen receptors are expressed in retinal ganglion cells (RGCs), and signaling is thought to have neuroprotective effects [13]. These findings indicate that estrogen has protective effects and is associated with a reduced risk of glaucoma.

We hypothesized that equol, which possesses estrogen-like effects and is derived from the diet, may have neuroprotective effects and may be useful as an addition to intraocular pressure-lowering treatment in glaucoma patients. Since it is clear that female hormones are related to glaucoma pathology, as mentioned above, this study aimed to investigate whether equol affects glaucoma.

## Methods

### Subjects

We prospectively studied 68 patients (26 male, 42 female; average age 63.0 ± 7.6 years) with an existing diagnosis of either unilateral or bilateral normal-tension glaucoma (NTG). We only included NTG patients to investigate non-IOP risk factors. The subjects were all Japanese and attended the glaucoma clinic at Tohoku University Hospital between February 2020 and March 2022. Control subjects were all patients with unilateral cataract who visited Tohoku University Hospital for cataract surgery and did not have glaucoma; the eye from each patient without cataract was included. When cataract was present in both eyes, we included the eye with better visual acuity.

Inclusion criteria were as follows: (1) if female, subjects were postmenopausal (i.e., menstruation was naturally absent, and the last menstrual period was at least 12 months before), and (2) no allergy to soy products. The exclusion criteria were (1) primary open-angle glaucoma, angle-closure glaucoma, exfoliative glaucoma, pigment dispersion glaucoma, any other type of secondary glaucoma, other ophthalmic conditions, or trauma, and (2) strong myopia (above 26.5 mm).

### Clinical parameters

We performed a complete ophthalmological examination of each subject, which included measuring best-corrected visual acuity (logarithm of the minimal angle of resolution; logMAR) and axial length; performing funduscopy, slit-lamp biomicroscopy, and gonioscopy; using Goldman applanation tonometry to measure intraocular pressure (IOP); using the Humphrey field analyzer (HFA; Carl Zeiss Meditec, Dublin, CA) to measure mean deviation (MD); and evaluating the optic disc with a 90-diopter lens. A glaucoma specialist performed all examinations.

In cases when the inclusion criteria were met by both eyes, we chose the eye with worse standard automated perimetry (SAP)-measured MD for inclusion in the statistical analysis. HFA examinations used the standard Swedish interactive threshold algorithm strategy of the 24–2 program.

### Equol measurement

Participants ate soy products that contained approximately 50 mg isoflavone (40–50 g of natto, 150–200 g of tofu, or 200 ml of soy milk). All participants provided first morning urine samples. Urinary levels of equol were measured with an immunochromatographic strip (Soy Check; Healthcare Systems) [14]. Participants were classified as equol producers based on a urinary equol level higher than 1.0 μM, as defined previously [15].

### Statistical analysis

We used the Spearman rank-correlation test. We used the Mann–Whitney U test and the Fisher exact test to compare groups. Multiple linear regression analysis was used to identify independent variables that affected MD. Numerical findings are reported as the mean ± SD, with statistical significance defined as *P* < 0.05. SPSS version 23.0 (SPSS Inc) was used in the statistical analysis.

## Results

**Fig. 1:**
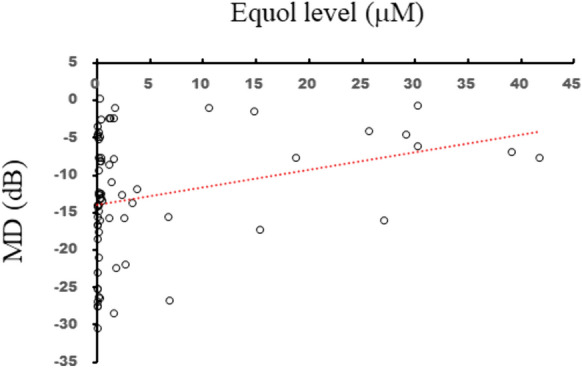
There was a significant association between equol producer status and MD (r = 0.36, *P* < 0.01)

**Fig. 2:**
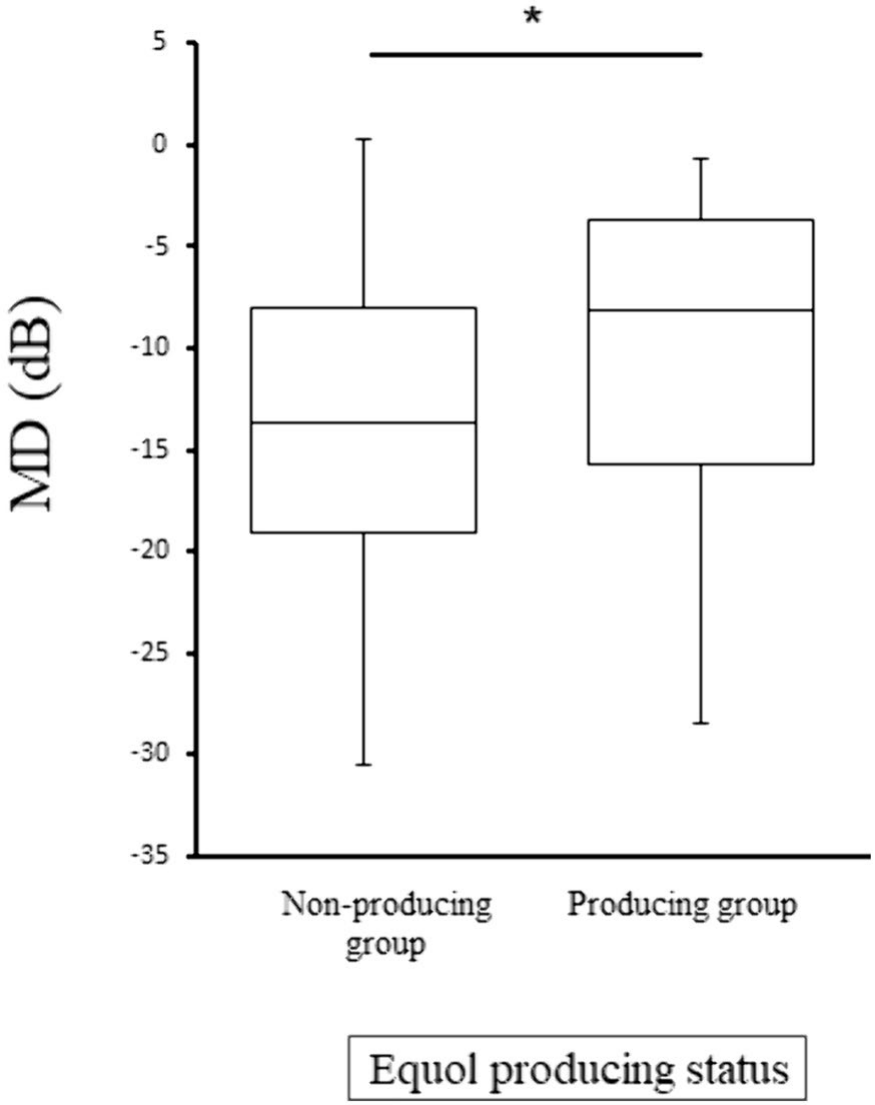
MD was higher among equol producers than non-producers. *P < 0.05

We recruited 31 controls and 68 NTG patients (Table 1). We observed significant differences in logMAR, axial length, and current smoker status between the controls and NTG patients (Table 1). Among the 68 patients with NTG, there were 28 equol producers (41.18%, Table 2). There was a significant difference between equol non-producers and producers in equol status (*P* < 0.01). Equol was significantly associated with MD in the 68 patients with NTG (r = 0.36, *P* < 0.01, Fig. 1). MD was significantly different in NTG patients who were equol non-producers and producers (*P* = 0.03, Fig. 2). Univariable regression analysis showed that equol level, age, IOP, and cpRNFLT independently contributed to MD (*P* = 0.01, *P* = 0.04, *P* = 0.03, and *P* < 0.01, respectively, Table 3). Multiple regression analysis showed that equol level, IOP, and cpRNFLT also independently contributed to MD (*P* = 0.03, *P* = 0.04, and *P* < 0.01, respectively, Table 3).

**Table 1:** Characteristics of control subjects and NTG patients

	Control	NTG	P value
Number	31	68	–
Male to female ratio	13:18	26:42	0.83^b^
Age (years)	66.0 ± 6.3	63.0 ± 7.6	0.07^a^
Equol (μM)	4.63 ± 8.29	4.91 ± 10.06	0.78^a^
Equol non-producer to producer ratio	16:15	40:28	0.52^a^
IOP (mmHg)	15.65 ± 1.82	13.32 ± 2.63	0.61^a^
Axial length (mm)	24.06 ± 1.34	24.82 ± 1.09	0.01^a^
LogMAR	0.26 ± 0.33	0.03 ± 0.27	< 0.01^a^
Hypertension (%)	15 (48.39)	27 (39.71)	0.52^b^
Diabetes (%)	6 (19.35)	11 (16.18)	0.78^b^
Hyperlipidemia (%)	12 (38.71)	22 (32.35)	0.49^b^
Heart disease (%)	4 (12.90)	6 (8.82)	0.72^b^
Current smoker (%)	17 (54.84)	21 (30.88)	0.02^b^
Body mass index (kg/m^2^)	23.37 ± 3.80	22.88 ± 3.17	0.54^a^

*IOP* intraocular pressure *logMAR* logarithm of minimum angle of resolution *NTG* normal-tension glaucoma

^a^Mann-Whitney test, ^b^Fisher test

**Table 2:** Patient backgrounds: equol non-producers vs producers

NTG patients	Equol non-producers	Equol producers	*P* value
Number	40	28	–
Male to female ratio	17:23	9:19	0.45^b^
Age (years)	63.5 ± 8.0	62.3 ± 6.9	0.45^a^
Equol level (μM)	0.20 ± 0.12	11.67 ± 13.10	< 0.01^a^
IOP (mmHg)	13.25 ± 2.74	13.44 ± 2.50	0.66^a^
Axial length (mm)	24.80 ± 1.14	24.84 ± 1.03	0.95^a^
LogMAR	0.02 ± 0.25	-0.003 ± 0.20	0.82^a^
Number of anti-glaucoma eyedrops	2.98 ± 0.23	2.86 ± 0.28	0.77^a^
Hypertension (%)	14 (35.00)	13 (46.43)	0.45^b^
Diabetes (%)	9 (22.50)	2 (7.14)	0.11^b^
Hyperlipidemia (%)	10 (25.00)	11 (39.29)	0.29^b^
Heart disease (%)	3 (7.50)	3 (10.71)	0.68^b^
Current smoker (%)	12 (30.00)	9 (32.14)	1.00^b^
Body mass index (kg/m^2^)	23.44 ± 3.21	22.07 ± 2.98	0.06^a^

*IOP* intraocular pressure *logMAR* logarithm of minimum angle of resolution *NTG* normal-tension glaucoma

^a^Mann-Whitney test, ^b^Fisher test

**Table 3:** Factors contributing to mean deviation in NTG patients

Variable	Univariable model		Multivariable model	
	β	*P* value	β	*P* value
Equol level (μM)	0.30	0.01	0.23	0.03
Age (years)	− 0.24	0.04	− 0.11	0.30
IOP (mmHg)	− 0.27	0.03	− 0.20	0.04
Axial length (mm)	− 0.09	0.48		
LogMAR	− 0.07	0.66		
cpRNFLT (μm)	0.60	< 0.01	0.51	< 0.01

*IOP* intraocular pressure *logMAR* logarithm of minimum angle of resolution *cpRNFLT* circumpapillary retinal nerve fiber layer thickness, *β* standard partial regression coefficient *NTG* normal-tension glaucoma

## Discussion

We found that equol producer status was significantly associated with glaucoma severity. Equol producers had significantly better MD than non-producers, and a multivariate regression analysis showed that equol was an independent contributor to MD. The estrogen-like action of equol is thought to have a neuroprotective effect. It is interesting that equol derived from soybean products synthesized by intestinal bacteria affected glaucoma pathology. This suggests that there is a relationship between diet and glaucoma. We believe that we are the first to make the finding that equol concentration correlates with glaucoma severity.

The relationship between estrogen and glaucoma has been examined in clinical, epidemiological, and basic research. Vajaranant et al. found that the early loss of estrogen caused by bilateral oophorectomy increased the risk of glaucoma development [16]. Basic research has reported that estrogen has neuroprotective effects. Nakazawa et al. found that RGC survival rate due to axonal damage changed before and after ovariectomy and that intraocular administration of estradiol activated ERK/cFOS in RGCs and exhibited neuroprotective effects [17], improved cell viability of Müller cells, and promoted brain-derived neurotrophic factor (BDNF) secretion [18]; these findings indicate that estrogen is important for neuroprotection. It has also been reported that estradiol weakens the activation of caspase 3, resulting in the prevention of apoptosis [19] and the suppression of oxidative stress [20]. These results suggest that estradiol should have protective effects on RGCs.

Equol binds to estrogen receptor β with strong affinity and has neuroprotective effects mediated by this receptor. Estrogen β receptors have been reported to be expressed in RGCs in the human eye [13]. According to this study, equol may have two effects. First, equol might exert a neuroprotective effect through estrogen receptor β in RGCs. Second, equol might act via the mitochondria, which studies have reported also express estrogen receptor β; activation of estrogen receptor β might regulate mitochondrial gene expression and anti-apoptosis effects [21]. Many previous reports show that there is a relationship between glaucoma and mitochondrial dysfunction [22]. Equol, as an estrogen receptor β agonist, might also moderate glaucoma pathology by improving mitochondrial function. We speculate that equol may have estrogen-like effects and may be neuroprotective in glaucoma pathology.

According to a report by Yoshikata et al., equol-producing bacteria were present in 97% of subjects studied, but only 22% of subjects had the ability to produce equol, indicating that there are many people who have equol-producing bacteria but do not produce equol [15]. Diverse intestinal bacteria are the key for equol-producing bacteria to function; it is important to actively consume vegetables, seaweed, root vegetables, mushrooms, and soybean foods. Non-equol-producing individuals have fewer intestinal bacterial strains, and have been reported to eat out more frequently, have irregular bowel movements, eat more ramen noodles, and smoke more. Therefore, in order to increase the ability to produce equol, it has been proposed that it is essential to modify dietary and lifestyle habits that regulate the intestinal bacterial environment. Interestingly, reports on lifestyle habits and glaucoma have shown that sleep [23, 24], diet [25, 26], and exercise [27] can moderate glaucoma, making it important to maintain a healthy lifestyle to prevent the progression of glaucoma. Equol exerts not only a neuroprotective effect through estrogen receptor β, but also an indirect effect via lifestyle habits. The ability to produce equol therefore represents a point of contact between lifestyle and glaucoma.

Sekikawa et al. measured blood equol levels in 91 cognitively normal elderly Japanese individuals, and after 6–9 years, measured the levels of white matter lesions and amyloid β deposits with brain imaging [28]. Their analysis showed that the blood concentration of equol did not affect the deposition of amyloid β, but revealed an association between equol and the reduction of white matter lesions. White matter lesions are a risk factor for cognitive decline. This result thus indicated that equol was a strong protective factor against the occurrence of white matter lesions. Glaucoma is also known to be a neurodegenerative disease. It is thus reasonable to consider that there may be a relationship between equol-producing capacity and glaucoma severity. Previous research has focused on intestinal bacteria and neuroprotective factors. For example, Gong et al. revealed there was a distinct difference in the composition of the gut microbiota and the serum metabolic phenotype between primary open-angle glaucoma patients and healthy individuals [29]. It has recently been shown that gut microbiota are important to the functioning of the central nervous system, that the microbiotic profile affects BDNF levels in the mouse hippocampus, and that reduced BDNF levels can be ameliorated by probiotic treatment [30]. It is possible that the microbiome could aid in glaucoma management via modulation of BDNF levels. It has been shown that BDNF has a potent protective effect in RGCs [31]; this led Gupta to conjecture that gut microbiota might increase neuroprotective factors, thereby promoting RGC survival [32]. Nevertheless, there is not yet direct evidence that the microbiome load or type affects retinal BDNF. Further research is needed to determine whether glaucoma pathophysiology is affected by the microbiome.

Limitations of this study include, first, a lack of estradiol measurements in the female patients. All female subjects were postmenopausal; we therefore considered that the male and female subjects would have similarly low estrogen levels and that our results would not be affected. Second, there was no difference in equol levels between the normal controls and the glaucoma patients; moreover, equol production was not associated with the onset of glaucoma. Nevertheless, there was a correlation between a patient’s equol status and the severity of their glaucoma, so we believe that equol status does affect the severity of glaucoma. Third, the number of enrolled patients was small, and no information on diet or on gut health were available. Such data, and a larger number of patients, will be necessary in the future. Fourth, the presence of more visual field defects in some patients may have indicated only that they had glaucoma for a longer time. Additional research and longitudinal data are needed to definitively determine the association between glaucoma and equol.

In summary, we conducted a study to measure the urinary level of equol, an isoflavone, in Japanese glaucoma patients. We found that patients who were equol producers had milder glaucoma than non-producers, suggesting that equol may contribute to the suppression of glaucoma progression.

## Conclusions

The severity of glaucoma was milder in equol-producing subjects than in non-equol-producing subjects, suggesting the possibility that equol is involved in suppressing the progress of normal-tension glaucoma.
